# A Recalcitrant Case of Senear-Usher Syndrome Treated With Rituximab

**DOI:** 10.7759/cureus.46910

**Published:** 2023-10-12

**Authors:** Jonathan M de Vries, Patricia Moody, Avaneesh Ojha, Alison Grise, Naveed Sami

**Affiliations:** 1 Internal Medicine, University of Central Florida College of Medicine, Orlando, USA; 2 Dermatopathology, KorPath, Tampa, USA

**Keywords:** senear-usher syndrome, desmoglein, bullous skin disease, rituximab therapy, superficial pemphigus

## Abstract

A 51-year-old uninsured, otherwise healthy male who works in the fishing industry presented with a two-month history of pruritic scaly plaques on his face, scalp, and trunk and mild photosensitivity. A biopsy of a scalp lesion revealed acantholysis consistent with pemphigus foliaceus. Laboratory testing demonstrated elevated anti-desmoglein 1, positive antinuclear antibodies (ANA and anti-dsDNA), and elevated Sjögren’s anti-SS-A antibodies. The patient was diagnosed with pemphigus erythematosus. The patient was not optimally responsive and was unable to discontinue systemic corticosteroids despite a maximum dosage of mycophenolate mofetil of 3000 mg/day. Hence, rituximab was added as a rescue treatment with the rheumatoid arthritis protocol. Three months after starting rituximab, there was a marked improvement in symptoms with complete resolution of cutaneous lesions.

## Introduction

Pemphigus erythematosus (PE), or Senear-Usher syndrome, is a type of superficial pemphigus exhibiting features of pemphigus foliaceus (PF) and systemic lupus erythematosus (SLE) [1]. It affects the face, scalp, and trunk with annular scaling plaques [2]. Diagnosis is based on clinical presentation, histopathology, immunofluorescence, and autoantibody serology [3]. Pemphigus erythematosus frequently presents in patients with other cutaneous autoimmune diseases, making clinical diagnosis more challenging [4]. Treatment includes systemic corticosteroids, dapsone, and immune-suppressing treatments. Rituximab is an established and successful therapy for different subtypes of acquired pemphigus [5]. However, there is little information available on the efficacy of this therapy or other biological treatments in treating PE [6]. There are few case reports on the successful usage of dupilumab, an IL4/IL13 inhibitor, including two cases of PE [7]. We report the case of a 51-year-old Latino male presenting with recalcitrant PE that was successfully managed with rituximab after failing to respond to high-dose mycophenolate mofetil (MMF) and being unable to decrease dosages of systemic steroids.

## Case presentation

**Figure 1:**
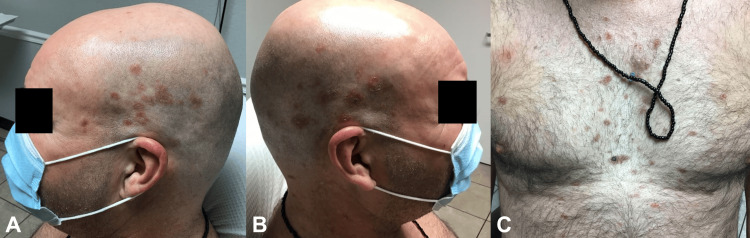
Initial presentation of scaling plaques on the head (A, B) and trunk (C)

A 51-year-old otherwise healthy Latino male presented with a two-month history of pruritic scaly plaques on the face, scalp, and trunk with accompanying photosensitivity. The patient also had erythema of the malar cheeks and nose. He was initially diagnosed with seborrheic dermatitis by a non-dermatologist provider. The condition was unresponsive and progressively worsened due to multiple oral antibiotic regimens (Figure 1). In addition, the patient complained of arthritis and Raynaud’s phenomenon.

A punch biopsy from a lesion on the right inframammary crease demonstrated findings more consistent with transient acantholytic dermatosis (Grover’s disease). The patient was prescribed topical triamcinolone (0.1%) and calcipotriene (0.005%).

**Figure 2:**
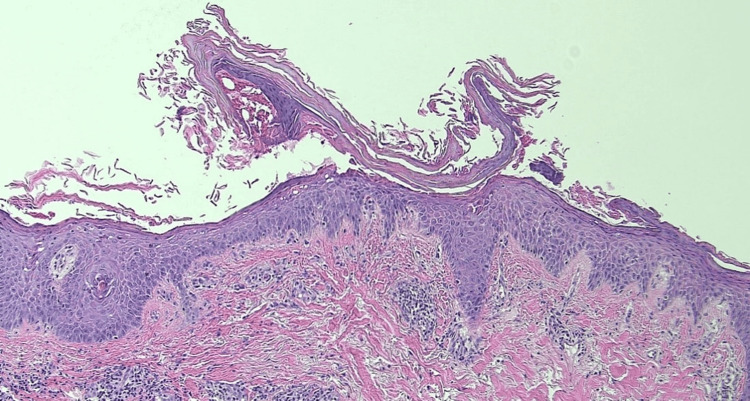
Punch biopsy of lesion showing subcorneal acantholysis and granular layer separation

One month later, the condition continued to progress and spread with superficial blistering and scaling plaques. An additional biopsy of the involved area on the posterior scalp revealed subcorneal acantholysis with foci of missing stratum corneum and granular layer separation (Figure 2). Direct immunofluorescence was not performed at the time of the biopsy due to a lack of insurance coverage.

Serologies were performed at a subsidized patient self-pay rate to confirm the diagnosis. Indirect immunofluorescence (IIF) revealed IgG (1:160) and IgG4 (1:40) intercellular antibodies on monkey esophagus. The serum enzyme-linked immunosorbent assay (ELISA) was positive for desmoglein 1 autoantibodies (151.4 ELISA units) and negative for desmoglein 3 autoantibodies (3.2 ELISA units). The patient was also positive for antinuclear antibodies (ANA 1:640), anti-dsDNA, and Sjögren’s anti-Ro (SSA) antibodies. An evaluation by rheumatology was negative for SLE. A diagnosis of PE was rendered based on the clinical presentation, histopathology, and serology results. The patient was initially treated with oral prednisone 20 mg/day and MMF 1000 mg/day.

After one month, the plaques regressed, but the patient reported mood changes that were not present prior to starting prednisone. Hence, the psychotropic symptoms seemed secondary to the systemic corticosteroids. The rheumatology consultation also confirmed these symptoms were likely induced by prednisone. A prednisone taper to 15 mg/day was initiated, and the MMF dose was increased to 2000 mg/day. The patient developed prednisone-induced folliculitis and continued to have intolerable psychological effects after six months. The patient was unable to taper below 5 mg/day of prednisone, and the MMF dose was increased to 3000 mg/day for an additional three months. Due to persistent cutaneous involvement, rituximab was considered and administered.

**Figure 3:**
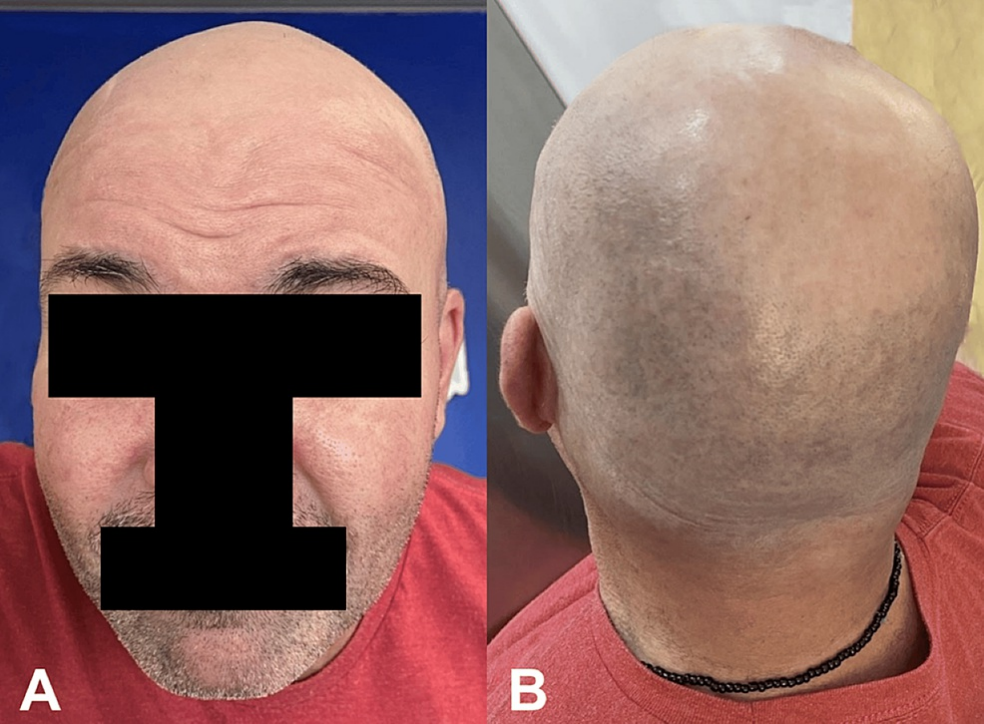
Complete resolution of lesions three months after initiation of combination therapy

The patient began rituximab infusions according to a modified rheumatoid arthritis protocol (1 g on day one and 1 g on day 15). Three months after the rituximab infusion, a marked improvement of symptoms with complete resolution of the PE lesions was noted (Figure 3). At the six-month post-infusion follow-up appointment, the patient had discontinued prednisone, and the MMF dose was tapered to 1500 mg/day.

## Discussion

Pemphigus erythematosus has been considered a subtype of PF. It presents with flaccid bullae leading to yellow-hued scaling plaques and hyperkeratosis similar to impetigo and other forms of pemphigus [4]. Lesions typically appear on sun-exposed cutaneous areas such as the head, neck, and upper trunk. Pemphigus erythematosus may also present in a malar distribution mimicking the facial-cutaneous presentation of SLE [5].

A diagnosis of PE can be confirmed with routine histology and direct and indirect immunofluorescence [8,9]. Serological studies for patients with PE are typically positive for anti-desmoglein 1 and/or anti-desmoglein 3, along with lupus antinuclear and anti-Ro/SSA antibodies [4,10]. 

Our patient was diagnosed with PE based on the clinical presentation and histopathological and serological studies. The patient was initially treated with systemic prednisone and MMF as a steroid-sparing agent. Due to adverse and persistent disease, rituximab was used as adjuvant rescue therapy. The patient exhibited a successful response and is now taking MMF 1500 mg/day as monotherapy.

Pemphigus erythematosus is often unresponsive to topical therapies and is treated with a combination regimen of oral corticosteroids, dapsone, and immunosuppressive agents such as MMF, methotrexate, azathioprine, and cyclophosphamide [5]. Patients whose disease control is systemic corticosteroid-dependent, unresponsive to oral corticosteroid-sparing medications, or intolerant to their adverse effects may benefit from rituximab. Rituximab is an FDA-approved treatment for pemphigus vulgaris [6].

## Conclusions

Pemphigus erythematosus is a rare autoimmune bullous disease with features of lupus erythematosus and PF. It can typically be treated with oral corticosteroids, MMF, and other immunosuppressant medications. Alternative treatments may be necessary for refractory cases or patients who cannot tolerate the effects of systemic corticosteroids. This case demonstrates rituximab as an alternative rescue treatment for PE. Further studies are needed to determine the efficacy of rituximab in treating PE.

## References

[1] Senear FE, Usher B (1926). An unusual type of pemphigus: combining features of lupus erythematosus. Arch Derm Syphilol.

[2] Gilman RL (1931). The Senear-Usher syndrome: a dermatosis combining features of lupus erythematosus and pemphigus. Arch Derm Syphilol.

[3] Amerian ML, Ahmed AR (1985). Pemphigus erythematosus. Int J Dermatol.

[4] Maize JC, Green D, Provost TT (1982). Pemphigus foliaceus: a case with serologic features of Senear-Usher syndrome and other autoimmune abnormalities. J Am Acad Dermatol.

[5] Pritchett EN, Hejazi E, Cusack CA (2015). Pruritic, pink scaling plaques on the face and trunk. Pemphigus erythematosus. JAMA Dermatol.

[6] Joly P, D'Incan M, Musette P (2007). Rituximab for pemphigus vulgaris. N Engl J Med.

[7] Chen J, Chen S, Wu X, Jiang X, Wang Y, Cheng H (2023). The complicated use of dupilumab in the treatment of atypical generalized pemphigus Erythematous: a report of two cases. Hum Vaccin Immunother.

[8] Pérez-Pérez ME, Avalos-Díaz E, Herrera-Esparza R (2012). Autoantibodies in senear-usher syndrome: cross-reactivity or multiple autoimmunity?. Autoimmune Dis.

[9] Chorzelski T, Jablońska S, Blaszczyk M (1968). Immunopathological investigations in the Senear-Usher syndrome (coexistence of pemphigus and lupus erythematosus). Br J Dermatol.

[10] Oktarina DA, Poot AM, Kramer D, Diercks GF, Jonkman MF, Pas HH (2012). The IgG "lupus-band" deposition pattern of pemphigus erythematosus: association with the desmoglein 1 ectodomain as revealed by 3 cases. Arch Dermatol.

